# IFN-γ response on T-cell based assays in HIV-infected patients for detection of tuberculosis infection

**DOI:** 10.1186/1471-2334-10-348

**Published:** 2010-12-10

**Authors:** Irene Latorre, Xavier Martínez-Lacasa, Roser Font, Alicia Lacoma, Jordi Puig, Cristina Tural, Josep Lite, Cristina Prat, Eva Cuchi, Vicente Ausina, Jose Domínguez

**Affiliations:** 1 Servei de Microbiologia. Hospital Universitari Germans Trias i Pujol. Fundació Institut en Ciències de la Salut Germans Trias i Pujol. Badalona. Spain; 2 Servei de Medicina Interna. Hospital Universitari Germans Trias i Pujol. Fundació Institut en Ciències de la Salut Germans Trias i Pujol. Badalona. Spain; 3 Unidad Clínica HIV. Hospital Universitari Germans Trias i Pujol. Fundació Institut en Ciències de la Salut Germans Trias i Pujol. Badalona. Spain; 4 Universitat Autònoma de Barcelona. Bellaterra. Spain; 5 Ciber Enfermedades Respiratorias. Instituto de Salud Carlos III. Badalona. Spain; 6 Unidad Control de la Tuberculosis/HIV. Hospital Universitari Mútua Terrassa. Spain; 7 Catlab. Terrassa. Spain

## Abstract

**Background:**

Individuals infected with human immunodeficiency virus (HIV) have an increased risk of progression to active tuberculosis following *Mycobacterium tuberculosis *infection. The objective of the study was to determine IFN-γ responses for the detection of latent tuberculosis infection (LTBI) with QuantiFERON-TB GOLD *In Tube *(QFT-G-IT) and T-SPOT.TB in HIV patients, and evaluate the influence of CD4 cell count on tests performance.

**Methods:**

We studied 75 HIV patients enrolled for ongoing studies of LTBI with T-SPOT.TB, QFN-G-IT and TST. Mean CD4 cell counts ± standard deviation was 461.29 ± 307.49 cells/μl. Eight patients had a BCG scar.

**Results:**

T-SPOT.TB, QFN-G-IT and TST were positive in 7 (9.3%), 5 (6.7%) and 9 (12%) cases, respectively. Global agreement between QFN-G-IT and T-SPOT.TB was 89% (κ = 0.275). The overall agreement of T-SPOT.TB and QFN-G-IT with TST was 80.8% (κ = 0.019) and 89% (κ = 0.373), respectively. We have found negative IFN-γ assays results among 2 BCG-vaccinated HIV-infected individuals with a positive TST. In non BCG-vaccinated patients, QFN-G-IT and TST were positive in 5 cases (7.5%) and T-SPOT.TB in 7 (10.4%). In contrast, in BCG-vaccinated patients, only TST was positive in 4/8 (50%) of the cases. The differences obtained in the number of positive results between TST and both IFN-γ assays in BCG vaccinated patients were significant (95% CI 3-97%, p = 0.046), however, the confidence interval is very wide given the small number of patients. In patients with CD4< 200, we obtained only one (5%) positive result with T-SPOT.TB; however, QFN-G-IT and TST were negative in all cases. On the contrary, percentages of positive results in patients with CD4> 200 were 10.9% (6/55), 9.1% (5/55) and 16.4% (9/55) with T-SPOT.TB, QFN-G-IT and TST, respectively.

**Conclusions:**

IFN-γ tests have the benefit over TST that are less influenced by BCG vaccination, consequently they are more specific than TST. Although our number of patients with advance immunosuppression is limited, our study suggests that IFN-γ assays are influenced with level of immunosuppression. The use of IFN-γ assays could be a helpful method for diagnosing LTBI in HIV population.

## Background

Tuberculosis (TB) is still a major cause of morbidity and mortality throughout the world. There is an estimated global incidence of 8.8 million new cases, with a total of 1.6 million deaths [1]. Indeed, individuals infected with human immunodeficiency virus (HIV) have an increased risk of progression to active TB following *Mycobacterium tuberculosis *infection of 5-10% per year [2]. The detection and treatment of active TB is crucial to control the global TB epidemic. Therefore, targeting and treating infected persons with high risk of disease reactivation is a key strategy for an effective control of the spread of TB.

Nevertheless, the diagnosis of latent tuberculosis infection (LTBI) is complicated due to the lack of a gold standard test. Tuberculin skin test (TST) has been used since the last century for diagnosing LTBI. TST measures a cell-mediated immunity as the form of a delayed-type hypersensitivity response to the purified protein derivative (PPD) [3]. The main drawback of the TST is its lack of specificity. PPD contains more than 200 antigens that are shared among other mycobacteria such as the Bacille Calmette-Guérin (BCG) vaccine strain and many non-tuberculous mycobacteria (NTM), consequently, false positive TST results can occur due to antigenic cross-reactivity [4]. In addition, the sensitivity of TST is reduced in HIV-positive patients because of false negative results, as a result of cutaneous anergy [5].

New *in vitro *T-cell based assays for the diagnosis of LTBI are now available. These assays measure the IFN-gamma (IFN-γ) released by sensitized T cells after specific *M. tuberculosis *antigen stimulation. These specific antigens are early secreted antigenic target 6 (ESAT-6) and culture filtrate protein 10 (CFP-10), which are encoded in the region of difference 1 (RD1) segment of *M. tuberculosis *genome [6], and TB7.7, encoded in RD11 segment [7].

Nowadays, there are two commercial available IFN-γ T-cell based assays: QuantiFERON-TB GOLD *In Tube *(QFN-G-IT, Cellestis Limited, Carnegie, Australia) and T-SPOT.TB (Oxford Immunotec Limited, Abingdon, UK). Both assays are approved from the U.S. Food and Drug Administration (FDA) as an aid for diagnosing LTBI. QFN-G-IT test stimulates whole-blood with ESAT-6, CFP-10 and TB7.7 in the same tube, and measures the concentration of IFN-γ in supernatants with an enzyme-linked-immunosorbent assay (ELISA). On the other hand, T-SPOT.TB assay stimulates isolated peripheral blood mononuclear cells (PBMCs) with ESAT-6 and CFP-10 separately, and detects number of IFN-γ producing T cells by means of an enzyme-linked immunospot assay (ELISPOT).

Promising results have been published with IFN-γ assays in the diagnosis of LTBI [8-13] and active TB [14-18]. Moreover, in the last years some studies have studied IFN-γ tests in HIV-infected population [19-23]. However, only few studies comparing the performance of both T-SPOT.TB and QFN assays in the same HIV population have been conducted [19,24-27]. However, more studies comparing T-SPOT.TB and QFN-G-IT with TST are required in order to better understand the role of IFN-γ assays in the diagnosis of LTBI in this kind of population, analyzing the impact of the degree of immunosuppression on the antigen-specific T-cell responses.

So, in the present study, we compared the utility of T-SPOT.TB, QFN-G-IT and TST for the diagnosis of LTBI in the same HIV population, and evaluated the influence of CD4 cell count on the different tests performance.

## Methods

### Study setting and patient recruitment

From January 2006 through November 2009, HIV-positive adults attending to the Hospital Universitari Germans Trias i Pujol and Hospital Universitari Mútua Terrassa were enrolled for ongoing studies of LTBI. The estimated TB community incidence of TB is 23.2/100.000 habitants and the HIV prevalence among those active TB patients is 8.5% [28].

Patients were consecutively recruited, and were enrolled during the course of the routine examinations. Each participant gave written informed consent before blood sampling. Ethics Committees of Hospital Universitari Germans Trias i Pujol and Hospital Universitari Mútua Terrassa approved the study. Information on the following variables was collected completing a detailed questionnaire: age, gender, BCG vaccination, prior TST (date and result), TB contact, history of prior active TB, chest radiography and other medical conditions. In our study, only participants with BCG scars were considered BCG vaccinated. In LTBI patients, active TB was excluded by clinical and radiologic examination. None of the patients included in this study had active TB. Patients were tested during the routine examination with the TST. Blood sampling of IFN-γ assays was performed before TST application. Patients with a previous documented positive TST were excluded.

### HIV testing and lymphocyte count

HIV testing was performed in all subjects. Blood samples were taken for HIV serology (ELISA and Western-Blot). CD4 and CD8 cell count were performed on blood samples from all HIV-positive patients.

### Tuberculin skin test

Two intradermal tuberculin units of PPD RT23 Tween 80 (Statens Serum Institut, Copenhagen, Denmark) were used to perform the TST, using the Mantoux method. Induration was measured 48-72 h after the application, and the size of the induration was interpreted by trained personnel. According to our national guidelines, an induration equal or higher than 5 mm was considered positive [29].

### QuantiFERON-TB GOLD *In Tube*

A total of three tubes of one millilitre each: nil control, positive control (phytohaemagglutinin [PHA]) and TB-specific antigens were drawn by venopuncture from each patient. The tubes were incubated overnight at 37°C, and after incubation plasmas were separated by centrifugation. The production of IFN-γ in whole-blood supernatant was determined by an ELISA.

Raw optical densities were interpreted by using specific software provided by the manufacturer. The result obtained by the nil control was subtracted from the positive control and the antigen-stimulated samples. The cut-off value for a positive test was at least 0.35 IU/mL of IFN-γ in the sample after stimulation with the specific antigens, regardless of the result of the positive control. The result of the test was considered indeterminate if the antigen-stimulated sample was negative and if the value of the positive control was less than 0.5 IU/mL after subtraction of the value of the nil control; and/or if the negative control was higher than 8.0 IU/mL.

### T-SPOT.TB

Eight millilitres of blood were drawn for the isolation of PBMCs in a vaccutainer CPT tube (Beckton Dickinson Diagnostics, Franklin Lakes, NJ). The isolated PBMCs were washed twice by centrifugation with RPMI medium (Invitrogen, Auckland, N.Z.), and later resuspended in AIM-V medium (Invitrogen, Auckland, N.Z.). Finally, viable cells were counted with an inverted microscope using the tripan blue method.

IFN-γ producing T cells were detected by an enzyme-linked immunospot assay (ELISPOT). The test was performed according the manufacturer's instructions. Each subject requires four wells precoated with a monoclonal antibody to IFN-γ. In the first well, cells were incubated with medium alone (control negative), in the second one with PHA (control positive), in the third one with ESAT-6 (Panel A), and in the last one with CFP-10 (Panel B). The assay requires a total of 250,000 cells per well.

On T-SPOT.TB, spots were scored using an automated AID ELISPOT plate reader (Lector AID Elispots, Autoimmun Diagnostiks GMBH, Germany). All readings were also manually verified. Each spot represents the footprint of a cytokine secreting cell and the number of spots obtained provides a measurement of frequency of *M. tuberculosis *sensitized cells. Subjects were considered positive if there was a positive response to one or both of the antigen panels. Test wells were scored as positive if they contained at least six spot-forming cells more than the nil control well and this number was at least twice the number of the nil control well. The result was considered indeterminate if the response to both antigen panels were negative and if the number of spots in the control positive well was less than 20. In addition, the immunoresponse was also considered indeterminate if the number of spots in the negative control was greater than 10.

### Statistical methods

Concordance between both tests was assessed using Cohen's Kappa (κ) coefficient. κ values below 0.40 indicate weak correlation, values of 0.41-0.60 indicate good agreement and values above 0.60 indicate strong agreement. Comparison of the number of spots and the IFN-γ released was performed by Mann-Whitney U test analysis. Differences were considered significant when *P *values were less than 0.05. All analyses were made with SPSS statistical software for Windows (SPSS version 15.0; SPSS Inc., Chicago; IL, USA). Graphical representation is based on GraphPad Prism version 4 (GraphPad Software, Inc, Dan Diego, CA).

## Results

### Patient characteristics

We studied 75 HIV-positive patients who were screened for LTBI. Mean CD4 and CD8 cell counts ± standard deviation were 461.29 ± 307.49 cells/μl and 899.33 ± 649.94 cells/μl respectively. The main demographic characteristics of patients included in the study are summarized in Table 1.

**Table 1:** Demographic characteristics of patients included in this study.

	All subjects N = 75 (%)	CD4< 200 N = 20 (%)	CD4 >200 N = 55 (%)
**Gender**			
*Male*	53 (70.7)	15 (75)	38 (69.1)
*Female*	22 (29.3)	5 (25)	17 (30.9)
**Age, mean ± SD**	42.41 ± 9.16	42.10 ± 7.96	42.53 ± 9.63
**BCG-vaccinated**			
*Yes*	8 (10.7)	2 (10)	6 (10.9)
*No*	67 (89.3)	18 (90)	49 (89.1)
**Birth country**			
*Immigrants from countries with high prevalence of TB infection*	6 (8)	1 (5)	5 (9.1)
*Autochthonous Spanish population*	69 (92)	19 (95)	50 (90.9)

### Diagnostic tests performance

The overall number of positive results in HIV-positive individuals screened for LTBI was 7/75 (9.3%), 5/75 (6.7%) and 9/75 (12%) using T-SPOT.TB, QFN-G-IT and TST respectively. There were not significant differences in the percentage of positive results between the three tests. We obtained two indeterminate results, both by T-SPOT.TB and QFN-G-IT, due to an insufficient response to PHA and *M. tuberculosis *specific antigens. In these two cases, TST was negative. Global agreement between T-SPOT.TB and QFN-G-IT was 89% (κ = 0.275; standard error [SE] = 0.184). The overall agreement of T-SPOT.TB and QFN-G-IT with TST was 80.8% (κ = 0.019; SE = 0.123) and 89% (κ = 0.373; SE = 0.173), respectively.

In non BCG-vaccinated patients, QFN-G-IT and TST were positive in 5/67 (7.5%), and T-SPOT.TB in 7/67 (10.4%) of the cases. In BCG-vaccinated patients both IFN-γ assays were negative, but in contrast, we obtained 4/8 (50%) of positive results with TST. The difference between the results obtained by TST in non BCG-vaccinated and BCG-vaccinated was statistically significant (p = 0.006). Furthermore, the differences obtained in the number of positive results between TST and both IFN-γ assays in BCG vaccinated patients were also significant (95% Confidence interval = 3-97%, p = 0.046), however, the confidence interval is very wide given the small number of patients. The number of positive results and the agreement between the assays regarding BCG-vaccination status are shown in Tables 2 and 3.

**Table 2:** T-SPOT.TB, QFN-G-IT and TST positive results regarding BCG vaccination status.

Diagnostic test	No. (%) of positive results		
	
	*BCG-vaccinated (n = 8)*	*Non BCG-vaccinated (n = 67)*	p*
**T-SPOT.TB**	0 (0)	7 (10.4)	0.531
**QFN-G-IT**	0 (0)	5 (7.5)	0.683
**TST**	4 (50)	5 (7.5)	0.006

*Significance value in percentage of positive results between BCG and non-BCG vaccinated patients.

**Table 3:** Concordance and agreement between TST, T-SPOT.TB and QFN-G-IT results according BCG vaccination status*

	TST vs T-SPOT.TB		TST vs QFN-G-IT		T-SPOT.TB vs QFN-G-IT	
	
	Concordance (%)	κ (SE**)	Concordance (%)	κ (SE)	Concordance (%)	κ (SE)
**HIV-positive**						
*Overall*	59/73 (80.8)	0.019 (0.123)	65/73 (89)	0.373 (0.173)	65/73 (89)	0.275 (0.184)
*BCG*	4/8 (50)	-***	4/8 (50)	-***	8/8 (100)	-***
*Non-BCG*	55/65 (84.6)	0.085 (0.154)	61/65 (93.8)	0.567 (0.194)	57/65 (87.7)	0.268 (0.186)

* Indeterminate IFN-γ assays results were not included in the analysis.

** standard deviation

***κ value is not possible to calculate because of T-SPOT.TB and QFN-G-IT results are one constant variable.

### Influence of CD4 cell count

**Figure 1:**
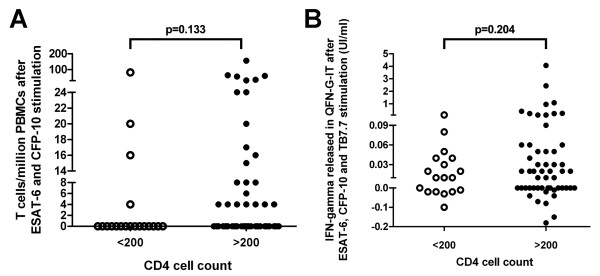
Number of responder T cells to specific antigens in T-SPOT.TB (A) and IFN-γ released after specific antigens (B) stimulation determined by QFN-G-IT in HIV-infected patients stratifying CD4 cell counts into two groups of < 200 and >200 cells/μl.

**Figure 2:**
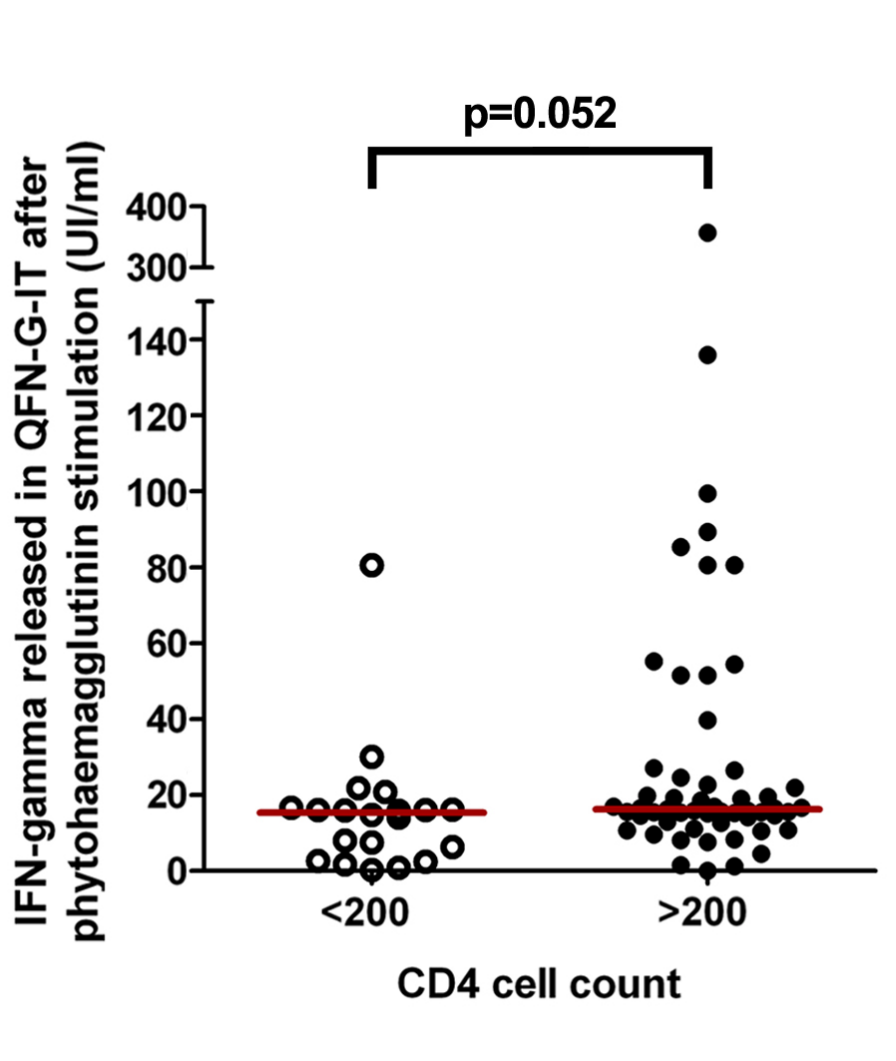
**IFN-γ released after PHA stimulation determined by QFN-G-IT in HIV-infected patients stratifying CD4 cell counts into two groups of < 200 and >200 cells/μl**.

We analyzed the possible impact of CD4 cell count on T cell responses, stratifying patients into two groups: 20 patients with < 200 CD4 cells/μl and 55 patients with >200 CD4 cells/μl. We found that number of responder T cells to specific *M. tuberculosis *antigens detected by T-SPOT.TB and the IFN-γ released in QFN-G-IT was lower in HIV-positive patients with CD4 cell counts < 200 than >200 cells/μl but not statistically significant, as shown in Figure 1. In addition, we studied the PHA T cell responses on QFN-G-IT according CD4 T cells and the differences between < 200 and >200 cell counts were nearly significant (Figure 2). On the other hand, it was impossible to asses the number of responder T cells after PHA stimulation on T-SPOT.TB due to saturation in the control positive well.

The proportion of positive results obtained by T-SPOT.TB and QFN-G-IT were lower in HIV patients with a CD4 cell count < 200 than above >200 cells/μl. In patients with a CD4 cell count below 200, we only obtained an only one (5%) positive result with T-SPOT.TB, that corresponded to one patient with 39 CD4 cells/μl. QFN-G-IT and TST were negative in all cases. In contrast, percentages of positive results in patients with a CD4 cell count above 200 were 10.9% (6/55), 9.1% (5/55) and 16.4% (9/55) with T-SPOT.TB, QFN-G-IT and TST, respectively. Differences in positive results regarding CD4 cell count were not significant for any tests (T-SPOT.TB, QFN-G-IT and TST: p = 0.313, p = 0.123 and p = 0.055, respectively).

**Figure 3:**
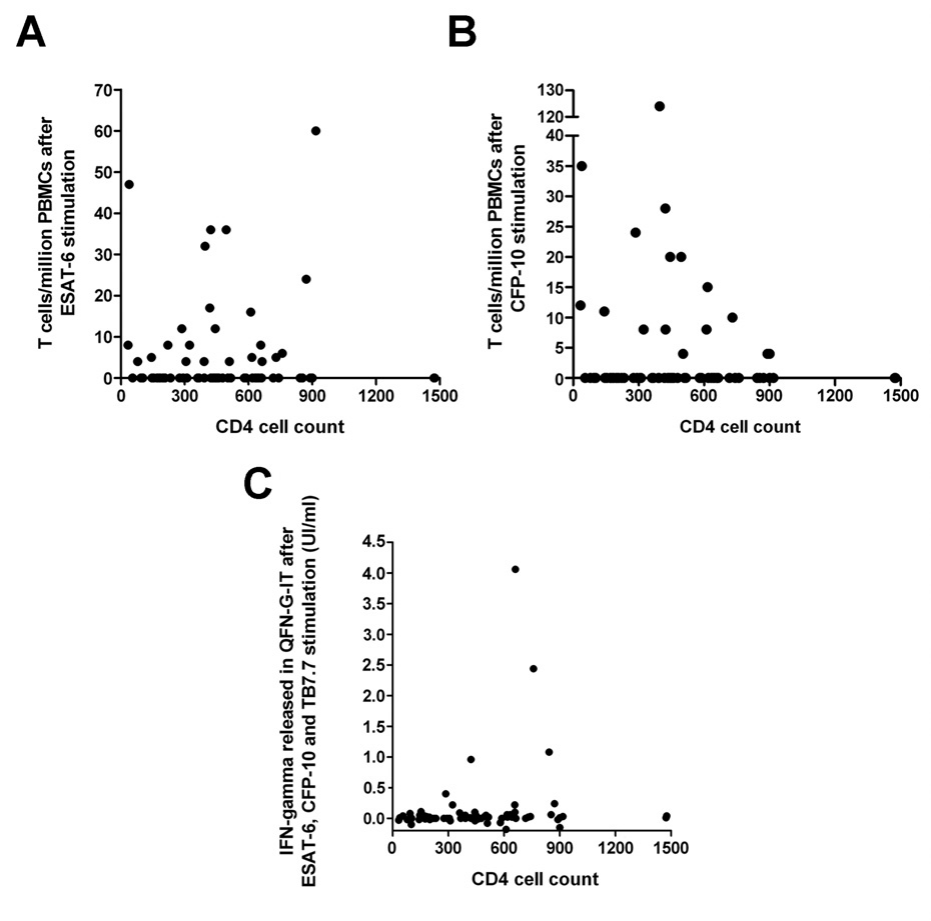
**Correlation of numbers of circulating CD4 T cells with responder T cells to ESAT-6 (A) and CFP-10 (B) in T-SPOT.TB, and IFN-γ released in QFN-G-IT (C)**.

The concentration of IFN-γ released in QFN-G-IT and the number of responder ESAT-6 and CFP-10 specific T cells detected by T-SPOT.TB was not correlated with number of circulating CD4 T cells (Spearman's rho [SR] = 0.221, p = 0.056; SR = 0.028, p = 0.813 and SR = 0.013, p = 0.910, respectively), as shown in Figure 3.

## Discussion

Several authors have studied the responses on T-cell based assays in HIV individuals for the detection of LTBI and active TB. It has been demonstrated in the majority of the studies, that IFN-γ assays have higher number of positive results than TST and a poor agreement with it [19-21,23,30-34]. Nevertheless, only a few studies have performed a direct comparison of T-SPOT.TB, QFN-G-IT and TST to ascertain LTBI in HIV-positive individuals [24-27].

Talati et al [25], compared T-SPOT.TB, QFN-G-IT and TST in 336 HIV-infected persons. They found a low prevalence of LTBI with the three diagnostic tests: 7 (2.1%) had a positive TST, 9 (2.7%) a positive QFN-G-IT and 14 (4.2%) a positive T-SPOT.TB. Overall, agreement between the three diagnostic tests was poor. Furthermore, Richeldi et al [24], performed simultaneously T-SPOT.TB, QFN-G-IT and TST in 116 chronically HIV-infected individuals. They identified a low percentage of individuals as LTBI and also a slight agreement between T-SPOT.TB and TST or QFN-G-IT. Rivas I [27] compared TST and both IFN-γ tests in 139 drug and alcohol abusers, 31% of them being HIV-infected patients. The authors did not found statistically significant associations between HIV serostatus and *in vitro *tests or TST. However, percentages of positive results obtained by T-SPOT.TB and QFN-G-IT in HIV-positive patients were higher for patients with a CD4 count >350 cells/μl than < 350 cells/μl (28.6% and 39.3% *versus *20% and 10%, respectively).

On the other hand, the evaluation of both T-cell based assays and the TST, in patients with HIV-infection, for the immunodiagnosis of LTBI has been also recently described in a high TB-incidence country. In this sense, Leidl et al [26] enrolled 109 individuals in Uganda with a new diagnosis of HIV-1 infection, and observed that global frequencies of positive results for TST, T-SPOT.TB and QFN-G-IT were 47.2%, 54% and 67.9% respectively. Although there are few differences between numbers of positive results in these previous findings, our results are consistent with those reported in the referred studies, where the commercial IFN-γ tests reached similar number of positive results. Furthermore, according to these studies we observed that concordances between three diagnostic tests were poor.

Intriguingly, we have observed poor agreement between both IFN-γ tests. From the 7 cases with a positive T-SPOT.TB result and the 5 cases with positive QFN-G-IT results, both tests were positive simultaneously in only 2 cases. Diagnostic agreement between tests was moderate (κ = 0.40-0.65). Similar results were described by Richeldi et al [24]. They observed that the agreement decreased in the HIV group when T-SPOT.TB was compared with either TST (κ = 0.16) or QFT-IT (κ = 0.19). In addition they reported highly discordant results (those clearly negative with one IFN-γ assay and clearly positive with another) in all groups of immunosuppressed patients. The analysis of these discordant results needs to be researched further.

It is poorly understood the impact of the HIV-infection in the immune response of LTBI and vice versa. We have observed, as other authors [24], the presence of discordant results between the TST and the IFN-γ tests, and also between both IFN-γ tests (T-SPOT.TB and QFN-G-IT). It is not clear enough the reasons for these kinds of results. In some cases it could be given by BCG vaccination, or by previous NTM infections (discordances between TST and IFN-γ tests). In other cases, it might be due to the different methodologies (TST *vs *QFN-G-IT *vs *T-SPOT.TB). In fact, maybe, the discordant results demonstrate some different immune responses [35], but this hypothesis has not been yet fully explored. In any case, given that a gold standard for LTBI does not exist, it is not possible to know, in case of discordant result, which test gives the true result. So, do we recommend doing TST and IFN-γ tests to all HIV patients? We think that we have to do more diagnostic effort in patients with a high risk of developing active TB if they are infected. Probably we have to use all the tests available in severe immunosuppresed patients, and maybe it is not so necessary in HIV patients with a conserved number of CD4. Therefore, the use of IFN-γ assays in combination with TST could be beneficial for diagnosing LTBI in HIV population severely immunosuppressed. However, probably our results did not provide definite data for supporting these comments.

We only detected two indeterminate results (2.7%) by either IFN-γ assays. This data is consistent with the majority of studies which show low rates of indeterminate results in HIV-infected patients without active TB [22]. One of the indeterminate cases obtained in our study corresponded with a low circulating CD4 cell count patient (103 cells/μl). It has been described that low CD4 cell counts are associated with IFN-γ assays indeterminate results for the diagnosis of LTBI and active TB [23,25,30,31,33,36,37]. However, in our study, 19 of 20 patients with < 200 CD4 cells/μl obtained a valid result. This data differ from other studies where high percentages of commercial IFN-γ tests or in-house ELISPOT assays indeterminate results were found in HIV-infected patients with low CD4 count cells in LTBI screening studies [23,25]. Nevertheless, studies of HIV-infected patients with active TB, generally report higher proportion of indeterminate results [36-38].

Raby et al [37] reported that low CD4 cell counts were associated with both indeterminate and negative QFN-G-IT results. On the contrary, low CD8 cell counts (median 369 cells/μl) were only associated with indeterminate results. They proposed that CD4+ and CD8+ cells could respond to PHA, unfortunately, the MCH class II overlapping peptides used in QFN-G-IT are only restricted to CD4+ cells. Consequently, those patients with high/normal CD8 cell counts (median 999 cells/μl) and low CD4+ cells produced a positive response to PHA but a negative response to *M. tuberculosis *specific antigens. In our study, only one of the two indeterminate results corresponded with a patient with a CD4 cell count < 200 cells/μl and a CD8 cell count < 600 cells/μl (103 and 568 cells/μl, respectively).

Regarding the differences in the IFN-γ assays results in HIV-positive individuals with a CD4 cell count < 200 respect those with a CD4 cell count >200, in our study all tests performed poorly in HIV-infected patients with CD4 cell count < 200. The fact that T-SPOT.TB obtained a positive result in one individual with a CD4 cell count < 200 (39 cells/μl), but none for QFN-G-IT, it is not sufficient to drawn conclusions. Converse et al [39], assessed the effect of HIV immunosuppression on QFN-G, and found that when immunosuppression increased, QFN-G sensitivity decreased, and only 30% (10/23) of HIV-seropositive persons with < 200 CD4 cells were positive.

Some studies have evaluated IFN-γ assays in HIV patients with active TB, and the influence of CD4 cell count. Aabye et al [36], reported that QFN-G-IT sensitivity in HIV-positive patients with active TB increased with high CD4 cell counts. Additionally, there are studies that have determined the diagnostic accuracy of T-cell based assays assessing the ratio of quantitative response of ESAT-6 and CFP-10 to CD4 T cell count, and improving the diagnosis of active TB [40,41]. No HIV-infected patient diagnosed of active TB was included in our study.

Furthermore, we observed that numbers of ESAT-6 and CFP-10 specific T cells in T-SPOT.TB, and concentration of IFN-γ in QFN-G-IT remained constant among patients with different levels of immunosuppression. Our results differ with those obtained in a recent study conducted by Leidl et al [26], where the correlation of the number of CD4 T cells with the IFN-γ released in QFN-G-IT was positive (Spearman's rho = 0.38; p = 0.0001), and constant with the number of ESAT-6 and CFP-10 specific T cells in T-SPOT.TB (Spearman's rho = 0.03; p = 0.77 and Spearman's rho = 0.13; p = 0.21, respectively).

Regarding the BCG-vaccination status, our results evidence that T-cell based assays are less influenced by BCG-vaccination than TST. In addition, we have found negative IFN-γ assays results among 2 non BCG-vaccinated HIV-infected individuals with a positive TST. A possible explanation to these discordant results could be a consequence of a previous NTM sensitization. In fact, in our experience, the utilization of IFN-γ tests could reduce the false diagnosis of LTBI in patients with a NTM sensitization [35,42]. In our study it was impossible to test *in vitro *NTM sensitins given that we didn't have more PBMCs stored from these patients. However, it is not clear enough the safety of not treating BCG and non BCG-vaccinated patients with a positive TST and negative T-cell based assays in this kind of population, especially in patients with more severe immunosuppression.

The main drawback of our study needs to be reported. Even though we have compared T-SPOT.TB, QFN-G-IT and TST in the same population of HIV-infected individuals, the number of patients, especially those with CD4 cell counts < 200, is limited. Furthermore, we did not detect any significant differences in the overall percentages of positive results between the three tests. However, our results reported in this study are consistent to add valuable data about the utility of the IFN-γ tests in the diagnosis of LTBI in HIV-infected patients, and the influence of the number of CD4 in the results. More studies comparing T-SPOT.TB and QFN-G-IT with TST are required to determine the role of IFN-γ assays for the diagnosis of LTBI in HIV-positive patients.

## Conclusions

In conclusion, IFN-γ tests have the benefit over TST that are less influenced by BCG-vaccination, consequently they are more specific than TST. The use of IFN-γ assays in combination with TST could be a helpful method for diagnosing LTBI in HIV population. Our study suggests that IFN-γ assays are influenced with level of immunosuppression. Further studies are required for understanding the meaning of the discrepancies between both IFN-γ tests.

## Competing interests

None of the investigators have any financial interest in or a financial conflict with the subject matter or materials discussed in this manuscript. None of the Scientific Societies, neither Inverness Medical Ibérica SAU (Barcelona, Spain), Cellestis (Carnegie, Australia) or Oxford Immunotec (Abingdon, UK) had a role in the study design, conduct, collection, management, analysis, or interpretation of the data, or preparation, review, or approval of the manuscript.

## Authors' contributions

*Conceived and designed the experiments: *IL, XM-L, VA and JD. *Performed the experiments: *IL, XM-L, RF, AL, JP, CT, JL, CP and EC. *Analyzed the data: *IL, XM-L, RF, AL, JP, CT, JL, CP, EC, VA and JD. *Contributed reagents/materials/analysis tools: *XM-L, CT, JL, CP and JD. *Wrote the paper: *IL, XM-L and JD. All authors read and approved the final manuscript.

## Pre-publication history

The pre-publication history for this paper can be accessed here:

http://www.biomedcentral.com/1471-2334/10/348/prepub
